# Association Between Heroin Use and Depression: NHANES 2005–2018

**DOI:** 10.1111/adb.70127

**Published:** 2026-01-21

**Authors:** Bei Li, Zhuojun Yang, Xiaoxiao Zhang, Mei Yang, Hong Qiu, Yulan Ren

**Affiliations:** ^1^ School of Acupuncture and Tuina Chengdu University of Traditional Chinese Medicine Chengdu China; ^2^ School of Chinese Classic Chengdu University of Traditional Chinese Medicine Chengdu China; ^3^ Sichuan Xinhua Compulsory Drug Rehabilitation Center Mianyang China; ^4^ Key Laboratory of Acupuncture for Senile Disease (Chengdu University of TCM) Ministry of Education Chengdu China

**Keywords:** cross‐sectional study, depression, heroin, mental health, NHANES, substance abuse

## Abstract

Heroin use and major depression are each leading contributors to global disability and premature mortality, yet evidence for a specific association between the two remains fragmented and is often derived from small, treatment‐seeking samples. We conducted a cross‐sectional analysis of nationally representative data from the 2005–2018 National Health and Nutrition Examination Survey, including 19 022 US adults aged ≥ 20 years with complete information on heroin use, depression status, and relevant covariates. Clinically significant depression was defined as a PHQ‐9 score ≥ 10. After multivariable adjustment for sociodemographic, behavioural, and clinical factors, including polysubstance use and chronic medical conditions, lifetime heroin use was independently associated with depression (adjusted OR = 1.85, 95% CI 1.43–2.40; *p* < 0.001). Subgroup analyses demonstrated that this association was robust and modified by age and smoking status, with significant interaction effects observed (*p* for interaction < 0.05). Restricted cubic spline analysis among participants with a history of heroin use (*n* = 439) revealed a non‐linear relationship between age at first heroin use and depression risk (*p* for non‐linearity = 0.032), with the highest predicted probability of depression among individuals who initiated use at or before 20.4 years of age. These findings indicate that lifetime heroin use is associated with a substantially increased risk of clinically significant depression in the general US population, particularly among younger adults and current smokers, underscoring the need for integrated screening and concurrent treatment of substance‐use and mood disorders.

## Graphical abstract

Graphical summary illustrating the association between lifetime heroin use and increased odds of clinically significant depression in US adults, with stronger effects observed among younger individuals and current smokers.

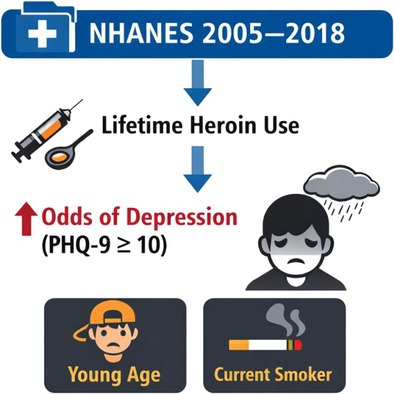

## 1 Introduction

Globally, heroin remains one of the most harmful illicit substances. The United Nations Office on Drugs and Crime (UNODC) estimates that 13.2 million people used heroin in 2022, and North America continues to face record‐high overdose deaths driven by synthetic adulterants [1]. Heroin use is associated with a cascade of adverse outcomes, including infectious diseases such as HIV and hepatitis C [2], neurocognitive impairment [3], unemployment, criminal involvement and all‐cause mortality rates that are 6–20 times higher than those of the general population [4].

Depression is a highly prevalent and disabling mental disorder worldwide, contributing substantially to the global burden of disease. The Global Burden of Disease Study ranks major depressive disorder among the top three causes of years lived with disability worldwide, with a 12‐month prevalence of approximately 5%–7% in adults [5]. Beyond individual suffering, depression predicts impaired occupational and social functioning, increased healthcare costs and a two‐ to three‐fold elevation in suicide risk [6].

Despite the individual public‐health impact of both conditions, compelling evidence for a specific association between heroin use and depression is still emerging rather than conclusive. Cross‐sectional data from the Australian Treatment Outcome Study showed that 54% of heroin‐dependent individuals met criteria for current major depression at intake, and depressive symptoms predicted poorer 12‐month retention and higher relapse rates [7]. A 2013 systematic review concluded that the relationship is bidirectional: Depression can accelerate the transition from recreational opioid use to dependence, while chronic heroin use, through neuroadaptive changes in reward and stress circuits, exacerbates or precipitates depressive episodes [8]. However, most prior studies were conducted in treatment‐seeking or incarcerated samples, relied on small sample sizes and rarely controlled for key sociodemographic and clinical confounders [3, 7, 9]. Consequently, whether observed associations reflect true causal pathways, shared vulnerability factors or ascertainment bias remains unresolved.

Using the nationally representative National Health and Nutrition Examination Survey (NHANES), the present study aims to quantify the cross‐sectional relationship between heroin use and depression in a general‐population sample, while adjusting for a comprehensive set of demographic, behavioural and medical covariates. Clarifying this association is essential to inform integrated prevention and treatment strategies that address both substance‐use and mood disorders concurrently.

## 2 Methods

### 2.1 Data Collection

This study drew its data from the NHANES, an open‐access repository maintained by the National Center for Health Statistics (NCHS). Employing a carefully structured, multistage probability design, NHANES gathers nationally representative information that encompasses sociodemographic descriptors, clinical examinations, biomarker assays and detailed self‐reports on diet and medical history. These components jointly yield a comprehensive snapshot of health conditions and lifestyle patterns across the US civilian population. The survey operates under strict ethical oversight, with informed consent obtained from every participant prior to data collection.

**FIGURE 1:**
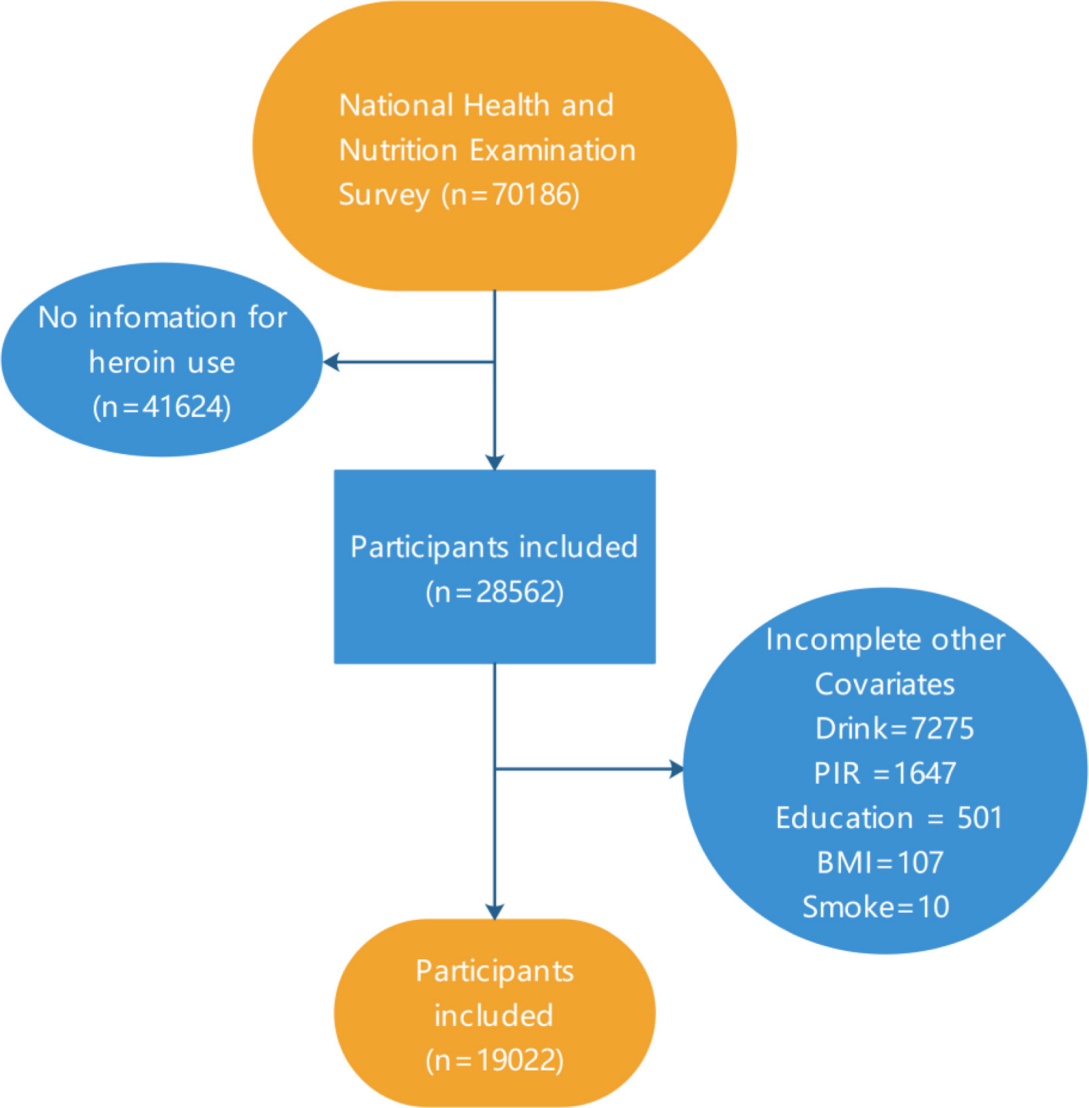
Flowchart of the sample selection from NHANES (2005–2018). BMI, body mass index; NHANES, National Health and Nutrition Examination Survey; PIR, poverty–income ratio.

Among the 70 186 participants included from NHANES 2005–2018, 37 706 participants lacked questionnaire data and were excluded. An additional 3918 participants were excluded due to missing heroin‐use information, leaving 28 562 participants. After further exclusion of participants with missing data on other covariates, 19 022 participants were included in the final analysis. The stepwise selection process is illustrated in Figure 1. All the data were obtained at https://wwwn.cdc.gov/nchs/nhanes/default.aspx.

### 2.2 Exposure Variable Measurement

Heroin use was defined as affirmative responses to both the general question (‘Have you ever used cocaine, crack cocaine, heroin, or methamphetamine?’) and the specific question (‘Ever used heroin?’).

### 2.3 Outcome Measurement

Depressive status was evaluated with the nine‐item Patient Health Questionnaire (PHQ‐9), a self‐administered scale aligned with DSM‐IV criteria for major depression [10, 11]. Respondents rated how often each of nine core symptoms had bothered them during the preceding 2 weeks on a four‐level frequency scale: 0 = *not at all*, 1 = *several days*, 2 = *more than half the days* and 3 = *nearly every day*. Individual item scores were totalled to produce a composite ranging from 0 to 27. Consistent with prior literature, a threshold of ≥ 10 was applied to classify participants as having clinically significant depression [12].

### 2.4 Covariates

Based on prior literature [13, 14], we included the following variables: age (20–34, 35–49 and 50+ years), education level (less than 9th grade, 9th–11th grade, high school graduate/GED, some college or AA degree and college graduate or above), race/ethnicity (non‐Hispanic White, non‐Hispanic Black, Mexican American, other Hispanic, non‐Hispanic Asian and other/multiracial), sex (male and female), body mass index (BMI), smoking status, alcohol status, diabetes mellitus (DM), anxiety and chronic pain. BMI was divided into the following four groups: underweight (< 18.5), normal (18.5–< 25), overweight (25–< 30) and obese (30 or greater). Smoking status was categorized into three groups: never smoked (< 100 cigarettes), former smoker (not currently smoking but ≥ 100 cigarettes previously) and current smoker (≥ 100 cigarettes and currently smoking every day or some days). Drinking status was classified as yes or no based on alcohol consumption of ≥ 12 occasions in the past 12 months. DM was diagnosed if participants were taking glucose‐lowering therapies, had an HbA1c concentration of ≥ 6.5%, used antidiabetic medications, had an oral glucose tolerance test result ≥ 11.1 mmol/L or a random blood glucose level ≥ 11.1 mmol/L. Anxiety and chronic pain were assessed based on participants' history of anti‐anxiety medication and analgesic use for ≥ 3 months.

Age, poverty–income ratio (PIR) and BMI were modelled as continuous variables.

### 2.5 Statistical Analysis

We conducted a two‐stage analysis.

Stage 1 (*n* = 19 022) estimated the overall association between lifetime heroin use and probable depression using survey‐weighted multivariable logistic regression.

Stage 2 (*n* = 439) examined whether age at first heroin use was non‐linearly related to depression. Because age at first heroin use was only available among participants with a history of heroin use, and cases with missing covariates were excluded, a total of 439 participants were included in the RCS analysis.

All data analyses were conducted in strict adherence to the Centers for Disease Control and Prevention (CDC) guidelines for NHANES statistical analyses. We selected the WTINT2YR variable as the weighting factor. The sample weights for individuals were calculated by WTINT2YR/7. Continuous variables were characterized by the mean and standard deviation (SD), while categorical variables were presented as sample counts and weighted percentages. To examine the variations in variable characteristics among the depression group, we utilized the Kruskal–Wallis test for continuous variables and the Rao–Scott chi‐squared test for the weighted percentages of categorical variables, thereby providing a comprehensive description of the entire population. To evaluate the relationship between heroin use and depression, we employed multiple logistic regression analysis. The results were reported as odds ratios (ORs) with 95% confidence intervals (CIs). Three models were constructed for the regression analyses. Model 1 included no adjustments. Model 2 adjusted for age, sex, race, PIR, education and BMI. Model 3 further adjusted for additional covariates, including smoking status, alcohol status, anxiety and chronic pain. Subsequently, we tested for interactions with all confounders in Model 3 and performed subgroup analyses. To assess the robustness of our findings, we conducted a sensitivity analysis by further adjusting the fully adjusted Model 3 for additional substance‐use variables—including methamphetamine, cocaine and marijuana use—as well as participation in drug rehabilitation programmes (defined as self‐reported participation in any drug treatment/rehabilitation programme). Statistical significance was established as two‐sided *p* < 0.05. Effect modification was assessed by including interaction terms between heroin use and candidate variables in the fully adjusted models. Variables with significant interactions (*p* for interaction < 0.05) were further examined in subgroup analyses. Subgroup analyses were conducted using pooled models by changing the reference category of the interacting variable, rather than by split‐sample analyses.

To test for a non‐linear association between age at first heroin use and probable depression (PHQ‐9 ≥ 10), we analysed the data (*n* = 439) by fitting a survey‐weighted logistic model that included restricted cubic splines (RCSs) with four knots (5th, 35th, 65th and 95th percentiles). The model adjusted for smoking status, age, sex, race/ethnicity, BMI, alcohol status, PIR, education, anxiety and chronic pain. Non‐linearity was evaluated with a Wald *χ* ^2^ test (3 df) comparing the spline terms to a linear‐only model; a two‐sided *p* value < 0.05 indicated significant non‐linearity.

All analyses were performed using R Version 4.4.3, employing various packages such as *dplyr*, *haven*, *gtsummary*, *forestplot*, *ggplot2*, *rms* and *survey*.

## 3 Results

### 3.1 Demographic Characteristics of Study Participants

Table 1 summarizes baseline characteristics by lifetime heroin‐use status. Of 19 022 participants, 581 (2.8%) reported having ever used heroin; 120 of these (19%) screened positive for depression (PHQ‐9 ≥ 10). Except for age, race, alcohol status, all other variables differed significantly between participants who did and did not report lifetime heroin use (*p* < 0.05). Participants with a history of heroin use, compared with those without such a history, had lower PIR, were more often male and non‐Hispanic White, had lower educational attainment and reported much higher levels of smoking consumption.

**TABLE 1:** Survey‐weighted characteristic variables of the study participants stratified by lifetime heroin use, US NHANES 2005–2018 (*N* = 19 022).

		Lifetime heroin use			
Characteristic	*N* ^a^	Overall, *N* = 19 022 (100%)^b^	No, *N* = 18 441 (97%)^b^	Yes, *N* = 581 (2.8%)^b^	*p* ^c^
Age (years)	19 022	42.9 (13.7)	42.9 (13.7)	43.0 (13.2)	0.8
Age.group	19 022				0.5
20–34 years		6180 (32%)	6029 (32%)	151 (32%)	
35–49 years		5915 (33%)	5754 (33%)	161 (30%)	
50+ years		6927 (35%)	6658 (35%)	269 (38%)	
PIR	19 022	3.16 (1.65)	3.19 (1.64)	2.40 (1.68)	< 0.001
PIR.group	19 022				< 0.001
3.5+		6592 (48%)	6465 (48%)	127 (31%)	
1.3–3.5		6810 (33%)	6628 (33%)	182 (33%)	
< 1.3		5620 (19%)	5348 (19%)	272 (36%)	
Sex	19 022				< 0.001
Female		8590 (47%)	8426 (47%)	164 (29%)	
Male		10 432 (53%)	10 015 (53%)	417 (71%)	
Race	19 022				0.12
Non‐Hispanic White		8464 (71%)	8166 (71%)	298 (74%)	
Non‐Hispanic Black		3964 (9.8%)	3816 (9.8%)	148 (11%)	
Mexican American		2963 (8.1%)	2901 (8.2%)	62 (6.3%)	
Other/multiracial		1863 (6.3%)	1834 (6.3%)	29 (4.2%)	
Other Hispanic		1768 (5.0%)	1724 (5.0%)	44 (4.6%)	
Education	19 022				< 0.001
Less than 9th grade		1194 (3.0%)	1160 (3.0%)	34 (4.5%)	
9th–11th grade		2504 (9.3%)	2390 (9.2%)	114 (15%)	
High school graduate/GED		4325 (22%)	4155 (22%)	170 (32%)	
Some college or AA degree		6173 (33%)	5961 (33%)	212 (39%)	
College graduate or above		4826 (32%)	4775 (33%)	51 (10%)	
BMI	19 022	29.02 (6.87)	29.06 (6.87)	27.69 (6.98)	< 0.001
BMI.group	19 022				< 0.001
Normal		5245 (29%)	5041 (28%)	204 (39%)	
Obese		7289 (37%)	7119 (37%)	170 (27%)	
Overweight		6213 (33%)	6017 (33%)	196 (33%)	
Underweight		275 (1.5%)	264 (1.5%)	11 (1.8%)	
Alcohol status	19 022				0.054
No		16 801 (87%)	16 307 (87%)	494 (82%)	
Yes		2221 (13%)	2134 (13%)	87 (18%)	
Smoking status	19 022				< 0.001
Current smoker		5125 (24%)	4779 (23%)	346 (55%)	
Former smoker		4527 (25%)	4346 (25%)	181 (35%)	
Never smoker		9370 (50%)	9316 (52%)	54 (9.3%)	
Chronic pain	19 022				< 0.001
No		16 635 (88%)	16 181 (88%)	454 (80%)	
Yes		2387 (12%)	2260 (12%)	127 (20%)	
Anxiety	19 022				0.002
No		17 884 (93%)	17 367 (93%)	517 (89%)	
Yes		1138 (6.6%)	1074 (6.5%)	64 (11%)	
Depression	19 022				< 0.001
No		17 233 (92%)	16 772 (92%)	461 (81%)	
Yes		1789 (8.0%)	1669 (7.7%)	120 (19%)	

Abbreviations: BMI, body mass index; NHANES, National Health and Nutrition Examination Survey; PIR, poverty–income ratio.

^a^ *N* not missing (unweighted).

^b^ Mean (SD) for continuous; *n* (weighted%) for categorical.

^c^ Design‐based Kruskal–Wallis test; Pearson's *χ* ^2^: Rao and Scott adjustment.

### 3.2 Association Between Heroin Use and Depression

As shown in Table 2, weighted logistic regression analysis found an association between participants with a history of heroin use and depression. Across Models 1–3, heroin use consistently demonstrated a significant positive association with depression. Their respective OR and 95% CIs were 2.82 (2.24–3.60), 2.40 (1.86–3.10) and 1.85 (1.43–2.40). The point estimates varied by ≤ 20%, and all 95% CIs excluded the null, indicating robustness of the observed relationship.

**TABLE 2:** Weighted logistic regression analysis models showing the associations between lifetime heroin use (*n* = 19 022) and depression (OR, 95% CI).

Depression	Model 1	*p*	Model 2	*p*	Model 3	*p*
No	Ref		Ref		Ref	
Yes	2.82 (2.24–3.60)	< 0.001	2.40 (1.86–3.10)	< 0.001	1.85 (1.43–2.40)	< 0.001

*Note:* Model 1: NO adjusted. Model 2: Adjusted for the variables in Model 1 plus age, sex, race, PIR, education and BMI. Model 3: Adjusted for the variables in Model 2 plus smoking status, alcohol status, anxiety and chronic pain.

Abbreviations: BMI, body mass index; PIR, poverty–income ratio.

### 3.3 Subgroup and Sensitivity Analyses

**FIGURE 2:**
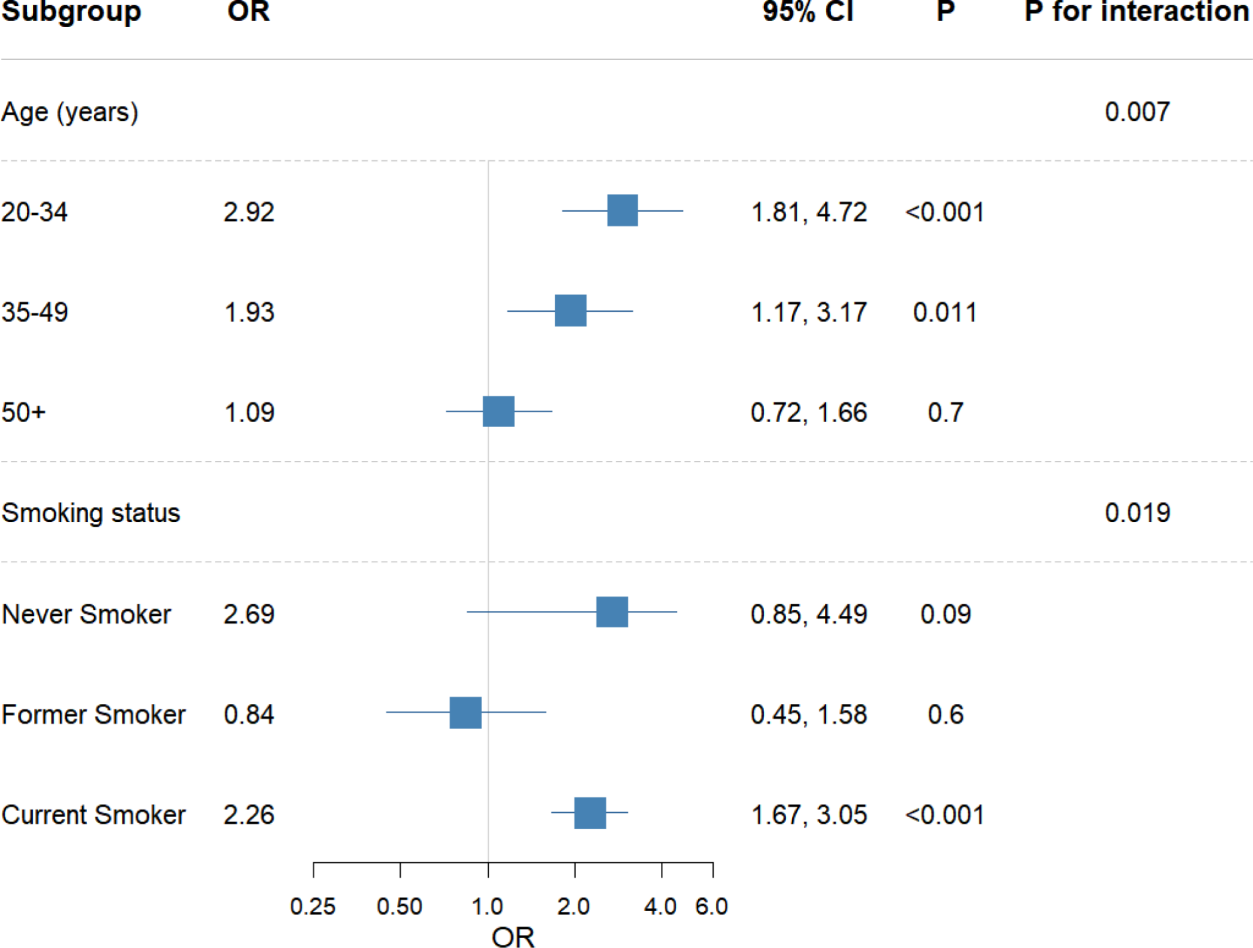
Association between lifetime heroin use (*n* = 19 022) and depression across subgroups. CI, confidence interval; OR, odds ratio.

Figure 2 presents the results of the subgroup and interaction analyses. Significant interactions were observed for age (*p* for interaction = 0.007) and smoking status (*p* for interaction = 0.019).

For the age subgroups, the association between lifetime heroin use and depression was strongest among participants aged 20–34 years (OR = 2.92; 95% CI: 1.81–4.72; *p* < 0.001), followed by those aged 35–49 years (OR = 1.93; 95% CI: 1.17–3.17; *p* = 0.011). The association was not statistically significant among adults aged ≥ 50 years (OR = 1.09; 95% CI: 0.72–1.66; *p* = 0.70). For smoking status, the association was not significant among never smokers (OR = 2.69; 95% CI: 0.85–4.49; *p* = 0.09) or former smokers (OR = 0.84; 95% CI: 0.45–1.58; *p* = 0.60) but was markedly stronger among current smokers (OR = 2.26; 95% CI: 1.67–3.05; *p* < 0.001). Interaction analyses of covariates with heroin use are shown in Table S1.

Table S2 shows the sensitivity analysis results. When additional adjustments were made in Model 3 for other drug use (methamphetamine, cocaine and marijuana) and participation in rehabilitation programmes, the association between lifetime heroin use and depression persisted and remained statistically significant.

### 3.4 The Non‐Linear Relationship Between Age at First Heroin Use and Depression

**FIGURE 3:**
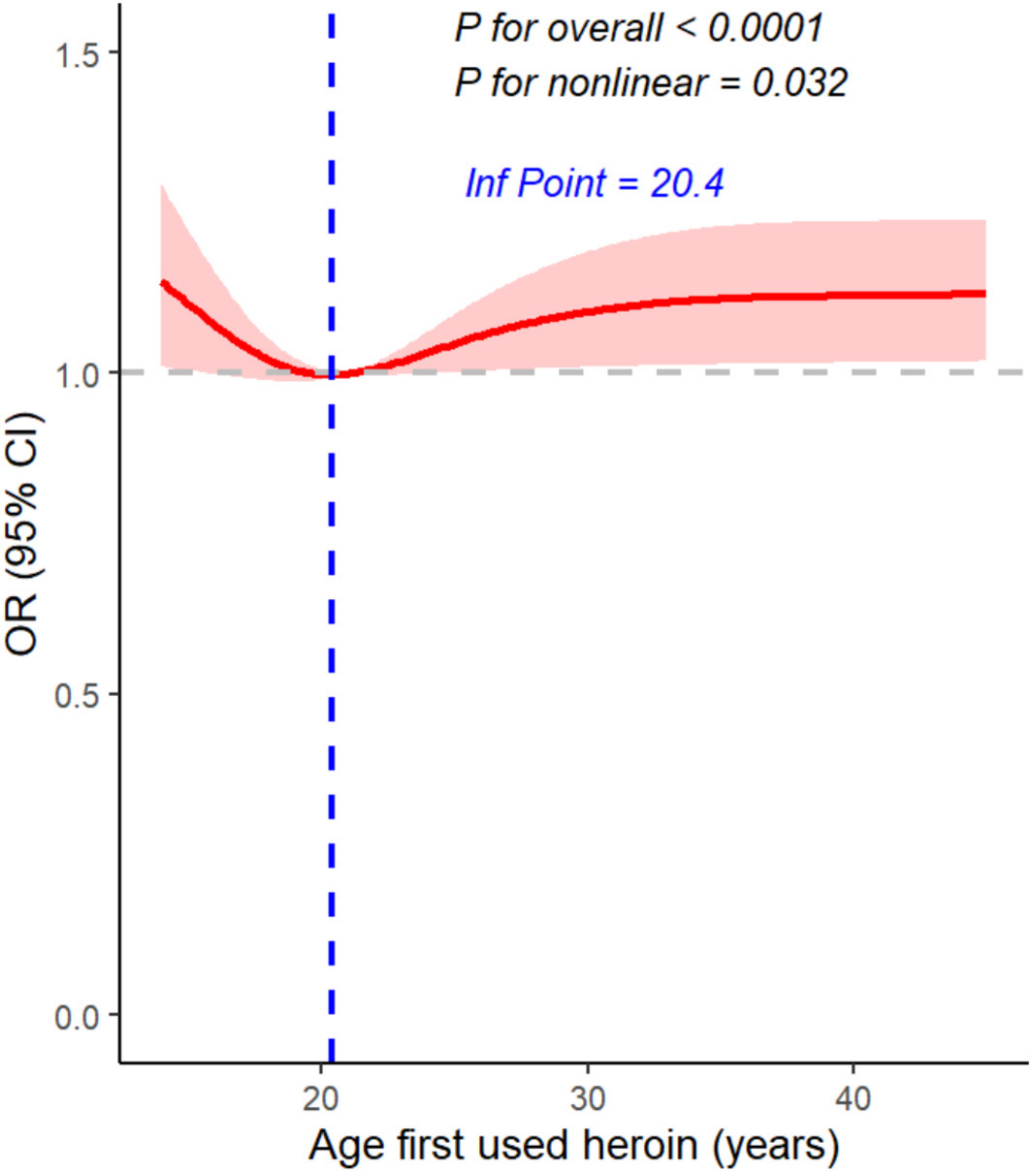
Restricted cubic splines reveal a non‐linear dose–response association between age at first heroin use (*n* = 439) and depression.

RCS (four knots, full adjusted) indicated a non‐linear association between age at first heroin use and the probability of depression (*p* for non‐linear = 0.032), with an inflection point at approximately 20.4 years (Figure 3). Sensitivity analyses using three and five knots yielded inflection points at 27.5 and 21.7 years, respectively, supporting the robustness of the four‐knot model (Figure S1).

## 4 Discussion

Using a nationally representative US sample (NHANES 2005–2018), we observed a robust cross‐sectional association between lifetime heroin use and clinically significant depression (PHQ‐9 ≥ 10). After comprehensive adjustment for sociodemographic, behavioural and clinical confounders—including polysubstance use and medical comorbidities—heroin use remained independently associated with an 85% increase in the odds of depression (adjusted OR = 1.85, 95% CI: 1.43–2.40). The consistency of subgroup and sensitivity analyses with the main results indicates the robustness of our findings. The relationship was strongest among young adults (20–34 years) and current smokers—populations that warrant intensified mental‐health screening and integrated interventions.

Our findings accord with converging preclinical and clinical evidence that participants with a history of heroin use and depression share dysregulation of mesolimbic reward and stress–response circuits. Chronic exposure down‐regulates μ‐opioid receptor signalling and attenuates dopaminergic tone in the nucleus accumbens [15, 16], while potentiating corticotropin‐releasing hormone activity in the amygdala—neuroadaptations that heighten anhedonia and stress reactivity, hallmarks of major depression [17, 18]. In addition, heroin‐related gut dysbiosis and systemic inflammation (increased IL‐6 and TNF‐α) may sustain depressive symptoms via blood–brain barrier disruption and microglial priming [19, 20]. The persistence of the association after extensive covariate adjustment suggests that these neurobiological mechanisms, rather than sociodemographic disadvantage or polysubstance exposure, account for a substantial share of the risk.

RCS revealed a modest non‐linear relationship between age at first heroin use and depression (*p* = 0.032). Sensitivity analyses using three and five knots placed the inflection at 27.5 and 21.7 years, respectively; the three‐knot model produced an almost flat curve, indicating oversmoothing. Overall, these findings point to initiation during adolescence or early adulthood as the period conferring the greatest risk of depression. Earlier initiation was associated with the highest predicted probability, supporting the view that exposure during key neurodevelopmental windows may create lasting vulnerability [21, 22, 23, 24].

Integrated care models that concurrently address opioid‐use and mood disorders have demonstrated superior outcomes compared with siloed treatment [25]. Screening for depression at every clinical contact with participants with a history of heroin use—and vice versa—could facilitate early detection and linkage to evidence‐based therapies (e.g., buprenorphine plus SSRIs or CBT) [26, 27]. Our stratified analyses further highlight priority subgroups: Young adults and current smokers exhibited the strongest associations, suggesting that preventive interventions targeting these demographics could yield maximal benefit [23]. Given the rising contamination of heroin with fentanyl analogues and escalating overdose mortality, embedding mental‐health services within harm‐reduction settings (syringe‐service programmes and supervised consumption sites) may be a pragmatic strategy [28].

This study combines clear strengths with unavoidable limitations. Strengths include the large, nationally representative sample, gold‐standard depression ascertainment (PHQ‐9) and comprehensive adjustment for confounders—including illicit polysubstance use rarely captured in electronic health records. Nonetheless, limitations merit consideration. First, the cross‐sectional design precludes temporal ordering; depression may precede and precipitate heroin initiation or vice versa. Second, heroin use was self‐reported, potentially introducing social‐desirability bias; however, prior NHANES validation studies suggest high concordance with urine toxicology [29]. Prospective cohorts leveraging repeated measures of both substance use and depressive symptoms are needed to disentangle directionality and to quantify the impact of depression remission on subsequent heroin‐use trajectories. Integration of neuroimaging, inflammatory biomarkers and genetic data within such cohorts could clarify mechanistic pathways [30]. Intervention studies should evaluate whether treating depression improves retention in medications for opioid‐use disorder (MOUD) and reduces relapse rates [31]. Third, given the rapidly shifting unregulated opioid market, real‐time surveillance systems that capture emerging synthetic adulterants will be critical to contextualize future epidemiologic findings [32]. Lastly, a substantial amount of missing data on heroin use may limit the generalizability of our findings.

## 5 Conclusion

In a broadly representative US sample, lifetime heroin use is independently associated with clinically significant depression. The association is robust across demographic and clinical subgroups, strongest among young adults and current smokers and only partially explained by shared risk factors. These data underscore the urgent need for integrated mental‐health and addiction services to mitigate the intertwined burdens of opioid use and depression.

## Author Contributions

**Bei Li:** conceptualization, writing – original draft. **Zhuojun Yang:** data curation, writing – review and editing. **Xiaoxiao Zhang:** formal analysis. **Mei Yang:** investigation. **Hong Qiu:** project administration. **Yulan Ren:** project administration, funding acquisition, supervision.

## Funding

This paper was supported by the Sichuan Provincial Administration of Traditional Chinese Medicine (No. 2024zd010). The funder had no role in study design, data collection and analysis, decision to publish or preparation of the manuscript.

## Ethics Statement

The survey operates under strict ethical oversight, with informed consent obtained from every participant prior to data collection.

## Conflicts of Interest

The authors declare no conflicts of interest.

## Supporting information


**Table S1:** Interaction effects between lifetime heroin use (*n* = 19 022) and other covariates on depression, US NHANES 2005–2018.
**Table S2:** Sensitivity analysis: supplementary sensitivity analyses with further adjustments using survey‐weighted logistics regression models, US NHANES 2005–2018.
**Figure S1:** Sensitivity analysis: restricted cubic spline plots using three knots (Panel A) and five knots (Panel B) for age at first heroin use (*n* = 439) and predicted probability of depression.

## Data Availability

The original contributions presented in the study are included in the article; further inquiries can be directed to the corresponding author.
