# Supplementary material for: Association Between Heroin Use and Depression: NHANES 2005–2018
Source: Addict Biol. 2026 Jan 21;31(1):e70127. doi: 10.1111/adb.70127 (PMC12820517; doi:10.1111/adb.70127)
Supplement: Supplementary file 1 — Table S1: Interaction effects between lifetime heroin use (n = 19 022) and other covariates on depression, US NHANES 2005–2018. Table S2: Sensitivity analysis: supplementary sensitivity analyses with further adjustments using survey‐weighted logistics regression models, US NHANES 2005–2018. Figure S1: Sensitivity analysis: restricted cubic spline plots using three knots (Panel A) and five knots (Panel B) for age at first heroin use (n = 439) and predicted probability of depression. [file ADB-31-e70127-s001.docx]

**Supplementary table S1 Interaction effects between lifetime heroin use (n = 19022) and other covariates on depression, U.S. NHANES 2005 - 2018**

| **Covariates** | **P for interaction** |
| --- | --- |
| Age | **0.007** |
| Sex | 0.9 |
| Race | 0.2 |
| PIR | 0.6 |
| BMI | 0.14 |
| Education | 0.8 |
| Smoking status | **0.019** |
| Alcohol status | 0.9 |
| Chronic pain | 0.2 |
| Anxiety | 0.8 |

Abbreviations: NHANES, National Health and Nutrition Examination Survey; PIR: poverty-income ratio; BMI, Body Mass Index.

**Supplementary table S2 Sensitivity analysis: supplementary sensitivity analyses with further adjustments using survey-weighted logistics regression models, U.S. NHANES 2005–2018**

| Depression | Use of other drugs(n = 18279) | | Use of other drugs+Reh(n= 10027) | |
| --- | --- | --- | --- | --- |
|  | OR(95%CI) | *P* | OR(95%CI) | *P* |
| No | ref |  | ref |  |
| Yes | 1.72(1.22, 2.41) | **0.002** | 1.68(1.19, 2.38) | **0.004** |

Sensitivity analyses were performed based on Model 3 and further adjusted by adding other drug use ([methamphetamine](https://wwwn.cdc.gov/Nchs/Data/Nhanes/Public/2013/DataFiles/DUQ_H.htm#DUQ330), cocaine, marijuana) and Reh, to assess the robustness of the model. Missing information has been excluded from the data. Results of adjusted OR, their 95% confidence intervals (95% CI), and P presented with bold values were statistically significant with P < 0.05 or P < 0.001.

Abbreviations: OR, Odds Ratio; CI, Confidence interval; NHANES, National Health and Nutrition Examination Survey; Reh, Rehabilitation.

**Supplementary Figure S1 Sensitivity analysis: restricted cubic spline plots using 3 knots (panel A) and 5 knots (panel B) for age at first heroin use(n = 439) and predicted probability of depression.**


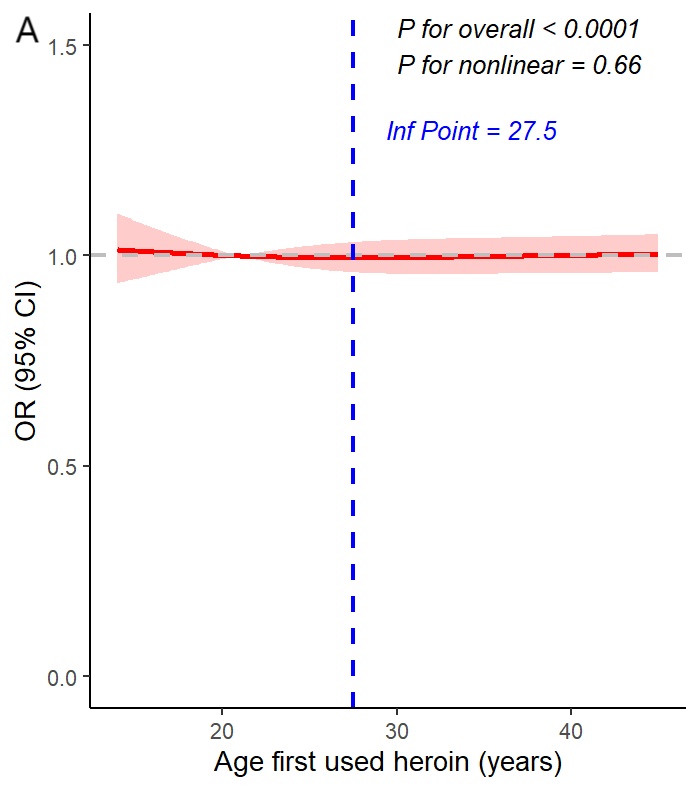

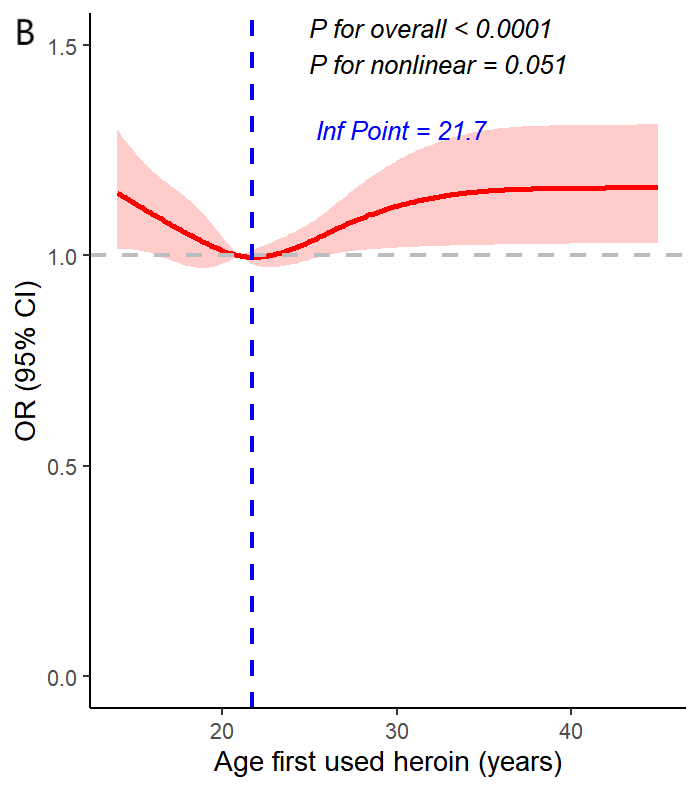


(A) 3-knot model: degrees of freedom were insufficient to capture curvature, resulting in an almost flat predicted probability curve and an inflection point at 27.5 years that is biologically implausible.
(B) 5-knot model: the fitted curve closely resembled the 4-knot model, with the inflection point shifting minimally to 21.7 years, indicating robustness of the non-linear relationship.
